# How weather impacts expressed sentiment in Russia: evidence from Odnoklassniki

**DOI:** 10.7717/peerj-cs.1164

**Published:** 2022-11-18

**Authors:** Sergey Smetanin

**Affiliations:** National Research University Higher School of Economics, Moscow, Russia

**Keywords:** Sentiment analysis, Natural language processing, Weather, Sentiment, Russia

## Abstract

Prior research suggests that weather conditions may substantively impact people’s emotional state and mood. In Russia, the relationship between weather and mood has been studied for certain regions—usually with severe or extreme climatic and weather conditions—but with quite limited samples of up to 1,000 people. Over the past decade, partly due to the proliferation of online social networks and the development of natural language processing techniques, the relationship between weather and mood has become possible to study based on the sentiment expressed by individuals. One of the key advantages of such studies based on digital traces is that it is possible to analyze much larger samples of people in comparison with traditional survey-based studies. In this article, we investigate the relationship between historical weather conditions and sentiment expressed in seven Russian cities based on the data of one of the largest Russian social networks, Odnoklassniki. We constructed a daily city-level expressed positive sentiment metric based on 2.76 million posts published by 1.31 million unique users from Odnoklassniki and studied its dynamics relative to daily weather conditions *via* regression modelling. It was found that a maximum daily temperature between +20 °C and +25 °C, light breeze (between 5 and 11 km/h) and an increase in the average daily temperature by 20–25 °C compared to the previous day are all associated with higher numbers of expressions of positive sentiment, whereas the difference between the maximum and minimum daily temperatures of 15–20 °C is associated with lower numbers of expressions of positive sentiment.

## Introduction

Although the existing body of knowledge clearly shows that weather affects individuals’ behaviour (Cohn, 1990; Deisenhammer, 2003; Parsons, 2001) and mood (Denissen et al., 2008; Howarth & Hoffman, 1984), studies investigating the association between weather and mood have found conflicting results (Keller et al., 2005). According to Baylis et al. (2018), this pattern of divergent results is due in part to a lack of large-scale data on emotional states, and one of the solutions is to leverage sentiment expressed in social media. Building on this line of research, several studies (Baylis, 2020; Baylis et al., 2018; Li, Wang & Hovy, 2014; Wang, Obradovich & Zheng, 2020; Zheng et al., 2019) have combined social media sentiment with weather conditions, using regression modeling to identify associations. However, considering that climate of the analysed region, time frame, and characteristics of the sample may have an impact on the identified associations between weather conditions and mood (Connolly, 2008; Denissen et al., 2008; Keller et al., 2005; Kööts, Realo & Allik, 2011; Parker & Tavassoli, 2000; Van de Vliert, Huang & Parker, 2004; von Mackensen et al., 2005), the investigation of the relationship between weather and mood in Russia is a relevant area of research.

Previous research (Feddersen, Metcalfe & Wooden, 2016) has shown robust evidence that day-to-day weather variation impacts self-reported subjective well-being, so the understanding of the magnitude of these relationships is essential for sociological, psychological and medical research. Although there have been several attempts (Hasnulin & Hasnulina, 2012; Lebedeva-Nesevria, Barg & Chechkin, 2020; Shiryaeva, 2015; Vesna & Shiryaeva, 2009) to study the relationship between weather and mood in Russia, these studies focused primarily on one particular region of Russian and had a very limited sample of up to 1,000 people. Moreover, such studies based on digital traces have not yet been conducted, thereby forming the gap in the body of research knowledge. However, it is worth noting that research has already been carried out in Russia that measures subjective well-being based on social media content (Kalabikhina et al., 2021; Panchenko, 2014; Shchekotin et al., 2020; Smetanin, 2022), thus laying the foundation for research on expressed sentiment and the weather.

In this work, we studied the relationship between historical weather conditions and sentiment expressed in social network Odnoklassniki. Firstly, we obtained a sample of posts from Odnoklassniki for 2 years and collected the weather conditions associated with them. Secondly, we automatically classified posts from Odnoklassniki into five sentiment classes using a pre-trained language model and built an Observed Positive Affect (Smetanin, 2022) indicator. Lastly, we applied regression modelling to investigate the relationship between weather and sentiment expressed in 2,764,468 posts by 1,312,408 million unique users, taking into account time and city effects. We found that a maximum daily temperature between +20 °C and +25 °C, light breeze (between $5\;km/h$ and $11\;km/h$), and an increase in the average daily temperature by 20–25 °C compared to the previous day are all associated with higher numbers of expressions of positive sentiment, whereas the difference in the maximum and minimum daily temperatures of 15–20 °C is associated with lower numbers of expressions of positive sentiment.

The rest of the article is organized as follows. In “Related Work”, we review the body of literature that studies the relationship between weather and expressed sentiment *via* social networks content. In “Data”, we describe the data sources for historical weather conditions, social network posts, and expressed sentiment. In “Analysis”, we propose the regression model for studying the relationship between weather and sentiment expressed in social network posts. In “Results and Discussion”, we present results of the regression analysis and discuss them. In “Limitations”, we discuss key considerations for the interpretation of obtained results. In “Conclusion”, we present conclusions from this study.

## Related work

In the past decade, an increasing number of studies have begun to address this problem through the analysis of big data, in particular through the analysis of the sentiment of geotagged social media posts. The core idea of such studies is to extract sentiment from social networks, couple it with weather conditions, and identify the association between them using regression modelling. For example, by examining two years of Twitter data, Li, Wang & Hovy (2014) discovered that mood state is sensitive to daily temperature change, daily precipitation, daily snow depth, and hail. Baylis et al. (2018) analysed 3.5 billion posts from both Facebook and Twitter and found that cold and hot temperatures, precipitation, narrower daily temperature ranges, cloud cover, and humidity are associated with worsened expressions of sentiment. The investigation of over one billion tweets by Baylis (2020) showed that in the United States there are consistent and statistically significant declines in expressed sentiment from both hot and cold temperatures. Zheng et al. (2019) constructed a daily city-level expressed happiness index based on the sentiment of 210 million posts from Sina Weibo and confirmed that air pollution, daily average temperature, wind, and rain have strong associations with expressed happiness. Wang, Obradovich & Zheng (2020) investigated the effect of weather extremes on expressed sentiment by coupling meteorological conditions with over 400 million social media posts from 43 million Chinese users of Sina Weibo and found that temperature, precipitation, cloud cover, and wind speed extremes are all correlated with more negative expressed sentiment. In the majority of mentioned studies, regression models with time and entity effects were used to study the relationship between expressed sentiment and weather conditions.

Thus, based on the analysed studies, we decided to (1) use regression modelling with time effects and entity effects to study the relationship between expressed sentiment and weather conditions and (2) consider the following weather conditions: maximum daily temperature, daily temperature change, the difference in average temperature compared to the previous day, daily snow depth, and wind speed. However, the final list of weather conditions was subsequently adjusted due to issues of data availability and completeness (see “Data” section). Moreover, we found no studies investigating the relationship between the weather and the sentiment of posts from social networks in Russian, which confirms the relevance of this study. Because previous studies have mostly relied on Twitter-like platforms, this is challenging in the context of analyzing Russian users because, firstly, Twitter is not very widespread in Russia (VCIOM, 2021b), and secondly, the existing open databases of Tweets, such as Twitter Stream Grab (https://archive.org/details/twitterstream), contain very few geotagged tweets in Russian.

## Data

### Social network posts

We used for our source of data posts in Russian from Odnoklassniki, one of the largest social networks in Russia (VCIOM, 2021b), with more than 40 million Russian users (Odnoklassniki, 2021). According to Brodovskaya, Dombrovskaya & Sinyakov (2016), Odnoklassniki is the most democratic social network in Russia because it is used by all categories of the population, including “traditional non-users”—that is, elderly and people with a low level of education. Also, as was reported by VCIOM (2018), the distribution of the Odnoklassniki audience by age is the closest among all social networks to the general distribution of the Internet audience in Russia.

The Odnoklassniki dataset consists of 8.6 million Russian-language text posts, which were published from Russia in a period from March 2019 to March 2021 by 3.3 million unique users. Each post contained an anonymized user identifier, the text, the time of publication, and the city in which the author was when the post was published. To the best of our knowledge, this is a largest available dataset of posts from Russia with information about users’ location. All user data was provided in the anonymized format; therefore, it was impossible to identify the real author of the post. The dataset was obtained from OK Data Science Lab (https://insideok.ru/blog/ok-data-science-lab/).

### Expressed sentiment

We classified sentiment of the obtained posts using fine-tuned RuRoBERTA-Large-RuSentiment (https://huggingface.co/sismetanin) published on HuggingFace within our previous study (Smetanin, 2022), which is a RuRoBERTa-Large model by Sberbank (2021) fine-tuned on RuSentiment (Rogers et al., 2018). RuSentiment is the biggest manually annotated dataset of general-domain VKontakte posts in Russian. Both VKontakte and Odnoklassniki are the most popular social networks in Russia (VCIOM, 2021a), very close in terms of the available functionality for communications (Blinova, 2013), and their posts are similar in terms of corpora similarity measure proposed by Dunn (2021), as was confirmed in Smetanin (2022). To the best of our knowledge, RuRoBERTA-Large-RuSentiment achieved the highest classification scores of weighted ${F_1} = 76.30$ and macro ${F_1} = 78.92$ (Smetanin, 2022) on RuSentiment and outperformed other pre-trained languages models, such as those studied in Baymurzina, Kuznetsov & Burtsev (2019), Kotelnikova et al. (2021), Kuratov & Arkhipov (2019), Smetanin & Komarov (2021).

The posts were divided into five classes: *Positive*, *Neutral*, *Negative*, *Speech Act*, and *Skip*. The detailed description of each class can be found in the original article by Rogers et al. (2018). As the measure of expressed sentiment, we decided to use the observable positive affect (Smetanin, 2022), which is calculated as follows:

(1) $$OSW{B^{PA}} = \displaystyle{{POS} \over {POS + NEG + NEU + SA + SKIP}},$$

where *POS* is the number of positive posts, *NEG* is the number of negative posts, *NEU* is the number of neutral posts, *SA* is the number of posts with greetings and speech acts, and *SKIP* is the number of ambiguous posts that cannot be unambiguously assigned to one of the other classes. This measure was selected because in our previous study on Odnoklassniki data (Smetanin, 2022) it demonstrated a high level of correlation with the survey-based VCIOM Happiness Index^1^ 1The VCIOM Happiness Index shows how happy Russians feel. VCIOM is one of the oldest Russian public research organizations that regularly conducts sociological and marketing research based on public opinion polls. (VCIOM, 2022) in Russia. Posts with speech acts were not considered as positive because they do not necessarily denote the the underlying positive sentiment of the author and may be expressed under social pressure (Dzogang, Lightman & Cristianini, 2018; Rogers et al., 2018). The number of negative posts is not used in the numerator because there is no evidence so far that it is correlated with happiness in the case of Odnoklassniki data (Smetanin, 2022).

**Figure 1:**
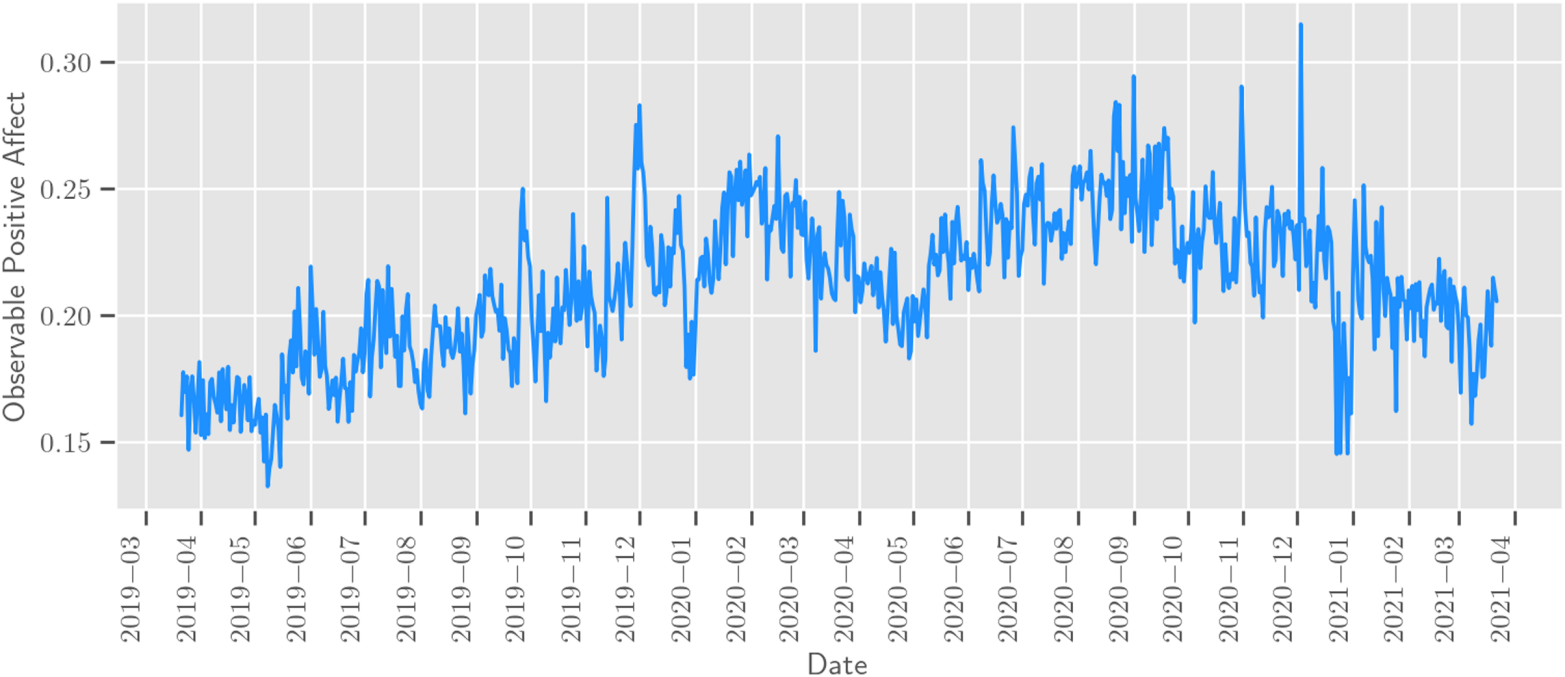
Observable positive affect in Moscow.

Before calculating the Observable Positive Affect metrics on a daily city level, we aggregated expressed sentiment on a daily city-user level to ensure that more active users are not over-represented^2^ 2As was highlighted by Németh & Koltai (2021), more internet-active people are more likely to appear in digital corpora due to the amount of posted messages. in our sample. When aggregating sentiment, we applied the majority rule: that is, we assigned each user the sentiment value that was most often encountered in his or her posts at the level of a certain day and city. Because in a significant number of cities there were only a few posts per day, we considered that the chosen metric in such cities could fluctuate greatly from day to day and negatively affect the quality of the analysis, so we decided to use only city-days with not less than 300 posts. Also, following the strategy by Wang, Obradovich & Zheng (2020), we kept only those cities that had enough posts on 60 or more days in the whole analysed time frame. As a result of the described filtering procedure, our final dataset consisted of 2,764,468 posts^3^ 3This number of posts is lower than the number of posts used in similar studies (e.g., Li, Wang & Hovy (2014), Baylis et al. (2018), Baylis (2020), Zheng et al. (2019), Wang, Obradovich & Zheng (2020)) focusing on other languages and countries. The main reason for having such a limited sample is that for the Russian language there are no other large public datasets of posts from social networks that contain information about the user’s location. published by 1,312,408 unique users from seven Russian cities: Moscow (1,216,691 posts), Yekaterinburg (391,852 posts), Krasnodar (376,529 posts), Novosibirsk (315,368 posts), Saint Petersburg (194,508 posts), Samara (143,677 posts), Rostov-on-Don (125,843 posts). An example of calculated Observable Positive Affect for Moscow can be found in Fig. 1.

### Weather conditions

The historical weather data used in this work was obtained through Meteostat (https://meteostat.net), an open weather and climate database providing detailed weather data for thousands of weather stations and locations worldwide. Meteostat uses weather and climate data provided by the following interfaces and organizations: Deutscher Wetterdienst, NOAA–National Weather Service, NOAA Global Historical Climatology Network, NOAA Integrated Surface Database, Government of Canada Open Data, MET Norway, European Data Portal, and Offene Daten Österreich.

**Figure 2:**
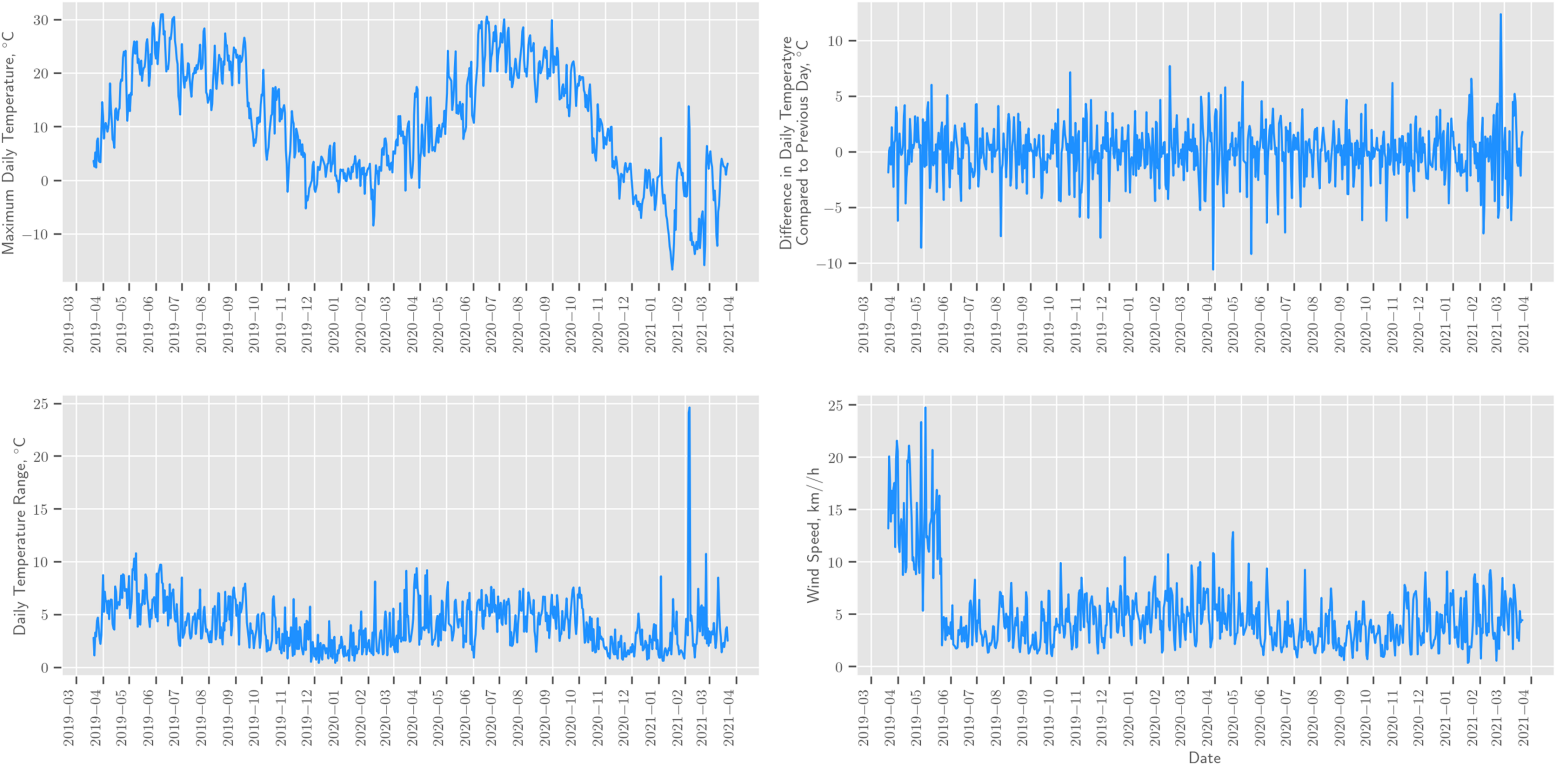
Weather conditions in Moscow obtained though Meteostat API.

Through Meteostat API, we were able to obtain data on weather conditions; however, we later found that the data on snow depth, humidity, precipitation, and cloud cover are not complete and often missing, so we excluded these weather factors from further analysis. An example of obtained weather conditions for Moscow can be found in Fig. 2.

## Analysis

In order to investigate the relationship between the weather and expressed sentiment, we combined aggregated city-level Observable Positive Affect scores with our weather data. We empirically modeled this relationship as follows:

(2) $$OSWB_{it}^{PA} = f(T_{it}^{Max}) + f(T_{it}^{TR}) + f(T_{it}^{TRPD}) + g\ (W{S_{it}}) + {\gamma _t} + {\alpha _t} + {{\epsilon }_{it}}$$

In this model, *i* indexes cities, *t* indexes calendar days, $OSWB_{it}^{PA}$ represents our Positive Affect measure of Observable Subjective Well-Being (further referred to as Observable Positive Affect) in the city *i* for the day *t*, and ${{\epsilon }_{it}}$ is the residual error of regression. Our independent variables of interest are the maximum temperature $T_{it}^{Max}$, temperature change within a day $T_{it}^{TR}$, the difference in average temperature compared to the previous day $T_{it}^{TRPD}$, and wind speed $W{S_{it}}$. We modelled temperature variables by using indicator variables (represented here by *f*()) for each 5 °C bin because such temperature bins allow for flexible estimation of the relationship between temperature and expressed sentiment (Baylis et al., 2018; Wang, Obradovich & Zheng, 2020). Also, we modelled the wind speed variable by using indicator variables (represented here by *g*()) for bins in accordance with the Beaufort wind force scale (Royal Meteorological Society, 2015). In order to avoid the Dummy Variable Trap, we omitted the 0 °C < *T^Max^* $\leq$ 5 °C maximum temperature bin, the 0 °C < *T_TR_* $\leq$ 5 °C temperature change within a day bin, the 0 °C < *T_TRPD_* $\leq$ 5 °C difference in average temperature compared to the previous day bin, and the gentle breeze ( $11\;km/h \le WS < 19\;km/h$) wind speed bin when estimating Eq. (2) (Baylis et al., 2018; Burke et al., 2018; Wang, Obradovich & Zheng, 2020). Therefore, the coefficients of interest could be interpreted as the effect on expressed sentiment change relative to these baseline categories. Since unobserved temporal or geographic factors may influence sentiment in ways that correlate with temperature and other weather variables (Baylis et al., 2018; Wang, Obradovich & Zheng, 2020), we also included in the model date fixed effects ${\gamma _t}$ and city fixed effects ${\alpha _t}$. ${\gamma _t}$ estimates the common change/difference (to all cities) in expressed happiness in date *t*, controlling for population density and city-specific time-invariant characteristics (the city fixed effects) (Buck, 2015). This variable is called a date fixed effect because the change is common to all cities in date *t*. In other words, the ‘effect’of date *t* is ‘fixed’ across all cities. ${\alpha _t}$ estimates the common change/difference (to all cities) in expressed happiness in city *i*, controlling for population density and date-specific characteristics/shocks common to all cities (the date fixed effects) (Buck, 2015). This variable is a city fixed effect because the change is common to all dates in city *i*. In other words, the ‘effect’ of city *i* is ‘fixed’ across all dates. Additionally, we clustered standard errors by cities.

Investigation of the relationship between weather and expressed sentiment for each city lies outside of the scope of this paper. As a result, our model was designed for analysis on the aggregated level. However, we suggest that this may be the actual direction for further research. It is worth noting that we assume that within the framework of this direction, researchers may encounter the following challenge. Different cities have different numbers of posts per day and different numbers of days suitable for the regression analysis, so city-level analysis may not necessarily be able to achieve statistically significant results for those variables for which the aggregate analysis showed statistically significant results. Moreover, in case of strong differences in weather conditions between cities, a situation may arise that in some city there are very few or no days with certain weather conditions (*e.g.*, in southern cities there are few days with a temperature below −25 °C). On the other hand, it is logical to expect that different variables may be statistically significant in different cities. In such a case, when comparing the results for different cities and the aggregated results, the question arises of how to interpret the difference between them, since it may arise both from a lack of data and from the fact that certain variables do have an effect in only certain cities.

## Results and discussion

**Figure 3:**
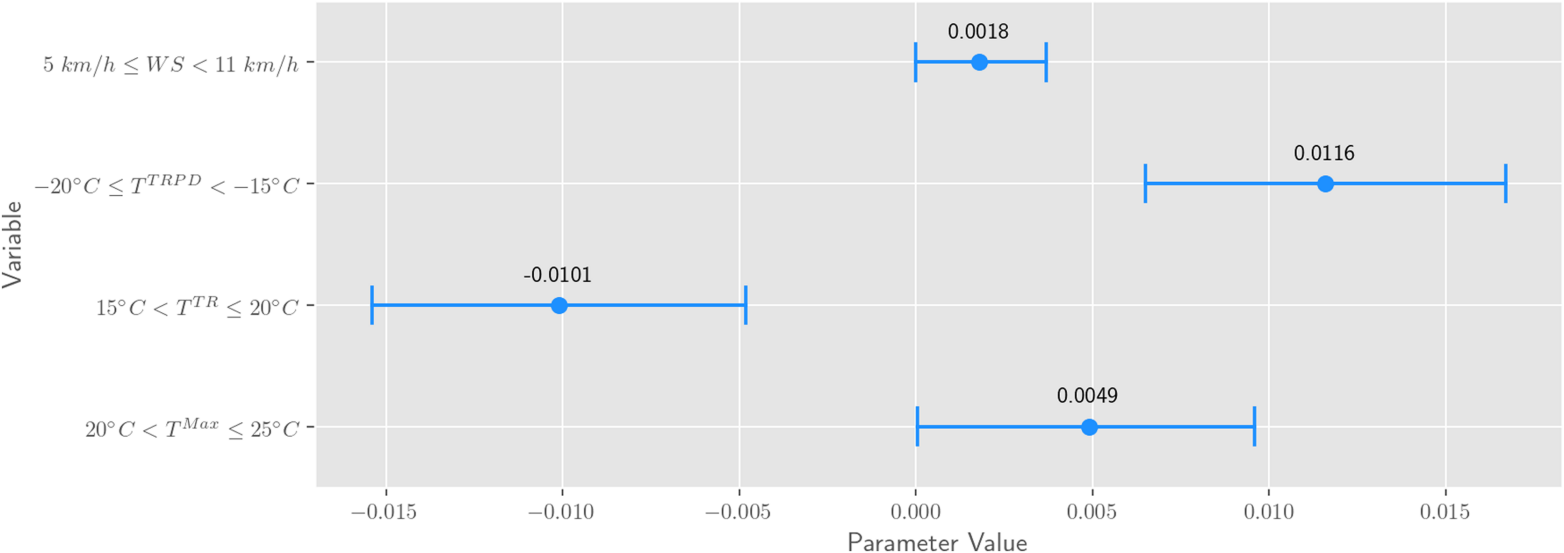
Statistically significant (*p* < 0.05) regression variables. The dot denotes the mean, the line denotes the confidence interval. The constant variable was omitted.

The results of estimating Eq. (2) for the association between expressed sentiment and weather on Odnoklassniki indicate that some bins of the wind speed, the difference in average temperature compared to the previous day, temperature change within a day, and maximum temperature have statistically significant associations with the expressions of positive sentiment. Statistically significant variables are presented in Fig. 3, and all variables^4^ 4Note that we can interpret only those coefficients that are statistically significant. are presented in Table 1.

- **Wind speed**. Light breeze ( $5\;km/h \le WS < 11\;km/h$) demonstrated statistically significant ( $p = 0.0495$) positive association with expressed positive sentiment. Considering that the gentle breeze ( $11\;km/h \le WS < 19\;km/h$) bin was a baseline, it can be interpreted as evidence that a weaker wind speed is positively associated with expressed positive sentiment, which is aligned with existing studies, such as Cooke, Rose & Becker (2000), Garcia (2021), Shan et al. (2021), Wang, Obradovich & Zheng (2020). All other wind speed bins showed no statistically significant association.
- **Daily temperature change**. The significant difference in the maximum and minimum daily temperatures of 15–20 °C is negatively associated with expressions of positive sentiment (the baseline bin is 0 °C < *T^TR^* $\leq$ 5 °C), suggesting that people prefer gradual temperature change rather than drastic. This finding is not entirely consistent with previous studies. For example, Baylis et al. (2018) reported that daily temperature ranges exceeding 15 °C are associated with significant increases in positive expressions, and Wang, Obradovich & Zheng (2020) did not find that daily temperature range has significant effects on sentiment. All other daily temperature change bins showed no statistically significant association.
- **Difference in average temperature compared to the previous day**. A strong warming of daily average temperature by 20–25 °C compared to the previous day is positively associated with expressed positive sentiment (the baseline bin is 0 °C < *T^TRPD^* $\leq$ 5 °C). This finding is not entirely consistent with previous studies. For example, Li, Wang & Hovy (2014) reported that people tend to feel uncomfortable with drastic temperature changes, and their responses to great temperature increase are unclear. All other bins showed no statistically significant association.
- **Maximum daily temperature**. The maximum daily temperature between +20 °C and +25 °C is positively associated with expressed positive sentiment (the baseline bin is 0 °C < *T^Max^* $\leq$ +5 °C). This finding is quite close to findings obtained through Twitter data by Baylis et al. (2018), in which the authors found positive expressions increase up to maximum temperatures of 20 °C. However, the authors also reported that positive expressions decline past 30 °C, which is not the case for our study. We hypothesize that this difference may have arisen because their study analyses a different region with different climatic conditions, and as was suggested by Parker & Tavassoli (2000), people in hot climates, compared to cold ones, present higher affective expressiveness, as well as higher sensitivity to emotional stimulation, both positive and negative. All other maximum daily temperature bins showed no statistically significant association.

**Table 1:** Regression parameters.

Variable bin	Parameter	*P*-value
*T^Max^* < −25 °C	0.0016	0.7971
−25 °C =< *T^Max^* < −20 °C	0.0077	0.1903
−20 °C =< *T^Max^* < −15 °C	0.0026	0.4841
−15 °C =< *T^Max^* < −10 °C	0.0028	0.2705
−10 °C =< *T^Max^* < −5 °C	−0.0002	0.9445
−5 °C =< *T^Max^* < 0 °C	0.0015	0.3411
*T^Max^* = 0 °C	0.0063	0.7660
+5 °C < *T^Max^* $\leq$ +10 °C	0.0015	0.3554
+10 °C < *T^Max^* $\leq$ +15 °C	0.0010	0.5879
+15 °C < *T^Max^* $\leq$ +20 °C	0.0041	0.0644
+20 °C < *T^Max^* $\leq$ +25 °C	0.0049	0.0475
*T^Max^* > +25 °C	0.0049	0.1024
5 °C < *T^TR^* $\leq$ 10 °C	−0.0003	0.7946
10 °C < *T^TR^* $\leq$ 15 °C	−0.0023	0.3610
15 °C < *T^TR^* $\leq$ 20 °C	−0.0101	0.0002
20 °C < *T^TR^* $\leq$ 25 °C	−0.0069	0.2467
−25 °C $\leq$ *T^TRPD^* < −20 °C	0.0109	0.0828
−20 °C $\leq$ *T^TRPD^* < −15 °C	0.0116	0.0000
−15 °C $\leq$ *T^TRPD^* < −10 °C	−0.0068	0.2811
−10 °C $\leq$ T^TRPD^ < −5 °C	0.0003	0.8687
−5 °C $\leq$ *T^TRPD^* < 0 °C	0.0008	0.2817
*T^TRPD^* = 0 °C	−0.0085	0.1261
5 °C < *T^TRPD^* $\leq$ 10 °C	0.0032	0.1040
10 °C < *T^TRPD^* $\leq$ 15 °C	0.0025	0.7290
20 °C < *T^TRPD^* $\leq$ 25 °C	0.0016	0.6093
$0\;km/h \le WS < 2\;km/h$ (calm)	0.0034	0.0940
$2\;km/h \le WS < 5\;km/h$ (light air)	0.0018	0.1938
$5\;km/h \le WS < 11\;km/h$ (light breeze)	0.0018	0.0495
$19\;km/h \le WS \le 28\;km/h$ (moderate breeze)	0.0009	0.5400
$28\;km/h \le WS < 38\;km/h$ (fresh breeze)	−0.0016	0.8697

Not all cases achieved complete agreement with existing studies; in our opinion, this is expected and generally aligns with a variety of findings presented in this area of research (see Barbosa Escobar et al. (2021)). First, other studies have been carried out for other countries or regions with different climates, and as a result, we can expect different associations. For example, Van de Vliert, Huang & Parker (2004) investigated data from 55 countries and found that people in low-income countries are less happy than the average of the 55 nations when temperatures deviate upwards or downwards from +24 °C, whereas people in high-income countries are happier when temperatures deviate upwards or downwards from +23 °C. Also, Parker & Tavassoli (2000) found that people in hot climates, compared to cold ones, present higher affective expressiveness. Secondly, these studies do not necessarily coincide with the time intervals for which the analysis was carried out, and, consequently, the obtained associations may be affected by seasonal factors. For example, Keller et al. (2005) found that higher temperatures improve mood during the spring because of the preceding lower temperatures during the winter, but during the summer, higher temperatures negatively affect mood. Lastly, other studies have been conducted on other groups of people, and the characteristics of these groups of people, in turn, may influence the resulting associations. For example, Denissen et al. (2008) suggested that the individual differences may have an effect on how people perceive the weather, von Mackensen et al. (2005) showed that older people are more sensitive to weather, Kööts, Realo & Allik (2011) found that older people tended to be somewhat differently affected by sunlight as well, and Connolly (2008) found that men responded to unexpected weather by simply changing their plans whereas women did not seem as likely to modify their activities, thereby more often taking the brunt of the unexpected weather on their mood.

## Limitations

There are several considerations important for interpreting the obtained results. Firstly, like other articles related to the study of the relationship between weather and expressed sentiment based on big data (*e.g.*, Baylis (2020), Baylis et al. (2018), Wang, Obradovich & Zheng (2020)), we have data on millions of posts from social networks, but we do not have access to the self-reported mood of these individuals. Although sentiment expressions on social media can be reflective of underlying emotions (Settanni & Marengo, 2015) and subjective well-being (Smetanin, 2022), there is still controversy in this area of research; further research is recommended to improve the psychometric validity of sentiment measures (Baylis et al., 2018). Secondly, the analysis presented in this study is conducted on users who self-select into participation in the social network Odnoklassniki; thus our results may not apply to demographics that do not use this particular social network or to the general population of analysed Russian cities. Thirdly, similar to Baylis et al. (2018), measurement error may exist between weather obtained through Meteostat API and the weather users actually experience, possibly impacting the magnitude of our estimates. Fourthly, the relationship between expressed sentiment and weather was observed from historical data, which might not persist into the future because of changing social and economic conditions, city environments, as well as policies (Wang, Obradovich & Zheng, 2020). Lastly, the model used for extracting sentiment from posts is not completely error-free, so the estimate of the relative occurrence of a particular class may be affected by misclassification bias, thereby affecting the value of the calculated index of expressed sentiment (Smetanin & Komarov, 2022).

## Conclusion

In this work, we studied the relationship between historical weather conditions and sentiment expressed in social network Odnoklassniki using regression modelling. We applied the sentiment classification model to 2,764,468 posts published by 1,312,408 unique users from seven Russian cities and calculated the Observable Positive Affect index for each city-day. The weather data was collected though Meteostat API. It was found that maximum daily temperatures between +20 °C and +25 °C, light breezes (between $5$ and 11 km/h) and increases in the average daily temperature by 20–25 °C compared to the previous day were all associated with higher numbers of expressions of positive sentiment, whereas the difference in the maximum and minimum daily temperatures of 15–20 °C is associated with lower numbers of expressions of positive sentiment. To the best of our knowledge, this is the first study dedicated to the relationship between weather conditions and expressed sentiment in Russian cities.

Further research might focus on several areas. Firstly, considerable work needs to be done to collect historical weather data for Russia, which includes complete information not only about temperatures and wind speed, but also about other weather conditions (*e.g*., humidity, air pressure, levels of sunlight, snow depth, precipitation), as well as conduct a more detailed analysis of relationship between weather and expressed sentiment. Secondly, it would be interesting to investigate this relationship for different demographic groups since previous research (Connolly, 2008; Denissen et al., 2008; Kööts, Realo & Allik, 2011; Van de Vliert, Huang & Parker, 2004; von Mackensen et al., 2005) suggests that weather may have different impacts on different groups of people. Thirdly, another possible area of future research would be to investigate the relationship between expressions of different emotions (*e.g.*, fear, anger, joy, sadness, contempt, disgust, and surprise) and weather conditions based on digital traces from Russia. Lastly, it would be interesting to analyse the relationship between weather and expressed sentiment on a city-level and compare this relationship between different cities.

## Additional Information and Declarations

### Data Availability

The following information was supplied regarding data availability: The data and code is available on GitHub: https://github.com/sismetanin/oswb-weather-odnoklassniki; Sergey Smetanin. (2022). sismetanin/oswb-weather-odnoklassniki: v1 (Version v1). Zenodo. https://doi.org/10.5281/zenodo.7232087.
